# Women’s experiences with yoga after a cancer diagnosis: a qualitative meta-synthesis—part I

**DOI:** 10.1186/s13643-023-02350-x

**Published:** 2023-09-26

**Authors:** Jenson Price, Sitara Sharma, Jennifer Brunet

**Affiliations:** 1 School of Human Kinetics, University of Ottawa, 125 University Private, Montpetit Hall, Room 339, Ottawa, ON K1N 6N5 Canada; 2 Cancer Therapeutic Program, Ottawa Hospital Research Institute, The Ottawa Hospital, Ottawa, ON Canada; 3 Institut du Savoir Montfort, Hôpital Montfort, Ottawa, ON Canada

**Keywords:** Mind–body, Oncology, Yoga, Female, Review

## Abstract

**Background:**

Qualitative research on women’s experiences participating in yoga after a cancer diagnosis is growing; systematic synthesis and integration of results are necessary to facilitate the transfer and implementation of knowledge among researchers and end-users. Thus, the purpose of this meta-synthesis was to: (1) integrate findings from qualitative studies, (2) compare and contrast findings to elucidate patterns or contradictions in conclusions, and (3) develop an overarching interpretation of women’s experiences participating in yoga after a cancer diagnosis.

**Methods:**

Using meta-study methodology, six electronic databases were searched using a sensitive search strategy in November 2020, a supplemental scan of reference lists was conducted in August 2021, and the database search was replicated in October 2021. Two reviewers independently screened titles/abstracts and full-texts to determine eligibility.

**Results:**

The searches yielded 6804 citations after de-duplication. Data from 24 articles meeting the eligibility criteria were extracted, and the results, methods, and theoretical approach(es) were analyzed. The analysis revealed that there was a predominant focus on two focal points in the primary articles: (1) women’s well-being and quality of life (QoL; part I) and (2) intervention preferences (part II). Five overarching categories emerged related to well-being and QoL: (1) yoga can support improvements in multiple dimensions of QoL in women diagnosed with cancer, (2) women diagnosed with cancer experience an interaction between QoL dimensions, (3) elements of yoga that support improvements in QoL dimensions, (4) breathwork and meditation are integral elements of yoga, and (5) yoga practice may support lifestyle behavior change. The articles reviewed had notable limitations related to: (1) reporting about instructor(s), content of the intervention, and environmental characteristics of the setting, (2) identifying and incorporating optimal features in the intervention design, (3) incorporating theory and real-world considerations into the study procedures, and (4) including positive and negative conceptualizations of QoL as an interconnected and multidimensional concept.

**Conclusion:**

Moving forward, it remains critical to identify the ideal structure and content of yoga programs for promoting well-being and QoL among women diagnosed with cancer, as well as to explore barriers and facilitators to sustainable program implementation.

**Systematic review registration:**

PROSPERO CRD42021229253.

**Supplementary Information:**

The online version contains supplementary material available at 10.1186/s13643-023-02350-x.

## Background

Globally, over eight million women are diagnosed with cancer each year [1], exerting tremendous physical, emotional, and financial strain on individuals, families, communities, and health systems [2]. The overall survival rate for cancer has improved in high-income countries due to accessible early detection, improved treatment, and better survivorship care. Similar to other high-income countries [3], the 5-year overall survival rate for women in Canada is 65% [4]. However, many who survive the disease report short- and long-term adverse effects that may be visible (e.g., scarring, deterioration of muscle mass and strength, reduced mobility [5, 6]) or non-visible (e.g., hot flashes, nausea, depression, anxiety, fear of recurrence, negative body image, cognitive dysfunction [7–9]. Indeed, due to symptoms and adverse treatment effects, women diagnosed with cancer have qualitatively reported reductions in their quality of life (QoL) and well-being, including feeling lost, uncertain, and angry about their situation and future [10, 11], as well as incapable of participating in their everyday lives with family, friends, and at work [7, 12]. Evidence suggests that QoL and well-being may be protective factors for health, reducing the risk of physical illness and promoting longevity among women diagnosed with cancer [13–16]; therefore, it is necessary to identify strategies to help women diagnosed with cancer self-manage unavoidable symptoms and treatment effects and ameliorate their QoL and well-being.

Definitions of QoL are diverse and can encompass a range of dimensions including physical (e.g., outcomes related to one’s ability to perform basic and instrumental physical activities related to daily living, leisure, and occupations), psychological (e.g., outcomes related to one’s thoughts, feelings, and self-perceptions), emotional (e.g., outcomes related to one’s emotional experiences and ability to manage their emotions), cognitive (e.g., outcomes related to mental processes involved in attention, language, perception, learning, memory, understanding, awareness, reasoning, and judgment), and social (e.g., outcomes related to one’s appraisal of their social relationships, how others react to them, and how they interact with social institutions and community) [17, 18]. In the oncology literature, QoL can be understood as a multi-dimensional concept that “refers to patients’ appraisal of and satisfaction with their current level of functioning compared to what they perceive to be possible or ideal” [19]. Previous research lends support to the view that greater well-being is associated with greater QoL and that QoL and well-being are separate concepts that can and should be fostered. Well-being refers to life evaluation (i.e., people’s thoughts about the quality or goodness of their lives), hedonic well-being (i.e., everyday feelings or moods as captured by both positive and negative adjectives), and eudemonic well-being (i.e., judgments about the meaning and purpose of life) [20, 21]. In this sense, well-being assessment carries unique information about a person’s status and enables development fundamental to QoL (and vice versa). Thus, the issue of maintaining both QoL and well-being in women diagnosed with cancer has become a key societal aspiration. Given the variable and expansive conceptualizations of QoL and well-being, the literature reviewed herein is discussed using a comprehensive lens and focuses on QoL as a global construct that encompasses well-being as a state of positive physical, psychological, emotional, cognitive, and social functioning that contributes to QoL.

Evidence has emerged that yoga can ameliorate QoL in women diagnosed with cancer [22–25]. Yoga is a form of complementary and alternative medicine [26] practiced for approximately 4000 years [27]. Although yoga originally evolved as a spiritual practice, its contemporary practice often features physical postures (asanas), breathing techniques (pranayama), and meditation (dhyana) in North America, Europe, and Oceania [28]. Among women diagnosed with cancer, several systematic reviews [22–25] and meta-analyses [29–32] have summarized the results of studies exploring yoga’s positive effects on outcomes such as health-related QoL, depression, anxiety, fatigue, and sleep disturbances. Notably, women diagnosed with cancer have also reported that yoga may help with self-management of symptoms and treatment effects by improving their capacity to cope with these [33–35] and reducing adverse physical effects (e.g., pain, numbness [36, 37]). While research on the underlying psychosocial mechanisms that may explain these positive effects is underdeveloped in the oncology field, research in the fields of body image and eating disorder suggest that the focus on moving, stretching, and balancing through a series of poses, awareness of breath, and cultivating the connection between mind and body may address both physical and psychological concerns. Specifically, the physical postures in yoga can be physically challenging or gentle, allowing participants to experience empowerment, strength, and/or relaxation, potentially facilitating greater connection to oneself with renewed attention on the body in a gentler, more compassionate, and positive manner [38–40]. Breathwork can provide a foundation for the calming of the mind through observation, control, or imagery [38–40]. Meditation can help participants meet their present-moment experience with openness, acceptance, and non-judgment [38–40]. However, it remains unclear whether these underlying mechanisms proposed to support QoL and well-being among other clinical and non-clinical populations translate to women diagnosed with cancer. Therefore, it is necessary to understand if and how physical postures, breathing, and meditation support QoL among women diagnosed with cancer.

Qualitative methods (e.g., interviews, focus groups) are optimal to gather knowledge on the benefits of yoga for women diagnosed with cancer and provide insight into *how* and *why* yoga is beneficial. Qualitative studies offer a consideration of the contextual information when observing and interpreting participants’ explanations and meanings of a phenomenon (i.e., a thick description [41]). In addition, qualitative studies attempt to document the complexity and multiplicity of individuals’ experiences [42]. Given that primary qualitative studies are rarely used on their own to contribute to practical knowledge [43] and that decision-making ought to be based on *all* the evidence available (i.e., quantitative and qualitative), methods to synthesize evidence from single qualitative studies have been developed to facilitate the transfer and implementation of knowledge emerging from qualitative studies. For example, qualitative evidence syntheses have been used to understand participants’ experiences, both comprehensively due to the qualitative approach and broadly due to the integration of studies from different contexts and participants, on various topics (e.g., fear of cancer in the general population [44]; experiences of adult children of parents with mental illnesses [45]). Despite a rise in the number of primary qualitative studies on women’s experiences participating in yoga after a cancer diagnosis, an analysis, synthesis, and interpretation of the collective findings is lacking.

Synthesizing results from qualitative studies relating to yoga for women diagnosed with cancer is central to understanding results more broadly and addressing important limitations of quantitative syntheses. Thus, a meta-study meta-synthesis using standardized, rigorous methods was conducted to synthesize the literature and address specific research questions about yoga for women diagnosed with cancer. The objectives of this meta-synthesis were to: (1) integrate findings from qualitative studies, (2) compare and contrast findings to elucidate patterns or contradictions in conclusions, and (3) develop an overarching interpretation of women’s experiences participating in yoga after a cancer diagnosis. Due to the extensive volume of data in the primary articles with two main focal points, it was necessary to present the findings in two distinct manuscripts to provide comprehensive and detailed insights for each outcome. The focal points naturally emerged from the in-depth analysis of the results/findings of the primary articles included in the meta-synthesis and were not set a priori. The current paper reports on findings focused on women’s well-being and QoL; it is part I. Part II reports on participants’ evaluations of yoga programs and interventions [46].^1^

## Materials and methods

The protocol for this review was registered in the International Prospective Register of Systematic Reviews (PROSPERO; registration number: CRD42021229253) and was published [47]. To complete this meta-study meta-synthesis, six distinct overlapping steps outlined by Paterson et al. [43] were undertaken (as described below). Reporting follows the enhancing transparency in reporting the synthesis of qualitative research (ENTREQ [48]) guidelines in lieu of Preferred Reporting Items for Systematic review and Meta-Analysis (PRISMA) guidelines as initially planned.

### Step 1: Formulating the research questions

Before conducting the literature search, two specific research questions were established: (1) What are the experiences of women who have participated in yoga after a cancer diagnosis? and (2) What elements of yoga contribute to participants’ positive or negative experiences?

#### Inclusion criteria

The inclusion criteria were set a priori and were broad to capture a breadth of experiences to better understand different responses to yoga. To be included, articles must have: (1) been primary studies conducted with women^2^ ≥ 18 years diagnosed with cancer, regardless of type of cancer, disease stage, and timing (e.g., at diagnosis, during treatment, post-treatment, or during palliative care), (2) used qualitative methods to collect data (e.g., interviews, focus groups, observations, journaling, open-ended survey questions), (3) reported on participants’ experiences engaging in yoga of any type and dosage (frequency, length, duration), and (4) comprised original research published in English language in a peer-reviewed journal. No restrictions were placed on the year of publication or study design (i.e., observational, quasi-experimental, experimental).

#### Exclusion criteria

Mixed-methods studies in which qualitative findings were not presented were excluded, as were gray literature (e.g., conference abstracts/posters/proceedings, unpublished theses/dissertations, websites, other unregulated sources), books, opinion pieces, and reviews. Moreover, studies with a sample consisting of > 50% men were excluded. This decision was made to ensure that the sampling method did not introduce bias in the analysis and interpretation of the entire corpus of data published [49, 50] and has been used in other health-related reviews focused on women’s experiences [51–53].

### Step 2: Selection and appraisal of the primary research

#### Step 2a: Systematically search and identify relevant articles

Articles were retrieved by searching six electronic databases: Medical Literature Analysis and Retrieval System Online (MEDLINE), Cumulative Index to Nursing and Allied Health Literature (CINAHL), PsycINFO, Scopus, SPORTDiscus, and Web of Science. With the help of a university librarian, a sensitive search strategy was developed by drawing on Medical Subject Heading (MeSH) terms and keywords used in published reviews (e.g., [54]). The chosen MeSH terms and keywords covered the population (i.e., women diagnosed with cancer) and terminology associated with yoga (e.g., yoga, mindfulness, physical postures, breathing exercises, meditation). To support the breadth of the search, the search strategy did not include MeSH terms and keywords covering data collection methods. The search strategy was pilot-tested and finalized in MEDLINE (see Additional file 1: Table S1) before being translated for use in the five other databases. To ensure the search strategy was compatible with the other databases, it was adjusted, as necessary, to reflect the varying syntax, indexing terms, and search functionalities of the databases while maintaining the core concepts and terms. An initial database search for articles was completed in November 2020, and the results were exported into Covidence, a systematic review online platform. A supplemental search of the reference lists of relevant articles retrieved during the electronic database search (i.e., reviews and included studies) was conducted in August 2021 to ensure all relevant articles were identified. The database search was replicated in October 2021 to retrieve citations published during the previous 11 months. A supplemental search of reviews and included studies was not conducted after the replication search due to the recency of the previous supplemental search. Covidence was used for the automatic removal of duplicates and to store, organize, and manage citations.

#### Step 2b: Study selection

After the removal of duplicate records, the titles and abstracts of the remaining citations were independently reviewed in a single step by two authors (JP and SS) using broad screening criteria (i.e., citations were not excluded if they did not explicitly state using qualitative methods or the gender/sex breakdown of the sample). This was followed by a full-text review of retained citations against the eligibility criteria; each full-text was independently reviewed by JP and SS. During both steps, the third author (JB) made the final decision when disagreements arose to avoid a hierarchy between screeners. Cohen’s kappa was calculated to measure inter-coder agreement at both screening steps and can be interpreted as follows: 0–0.20 = none, 0.21–0.39 = minimal, 0.40–0.59 = weak, 0.60–0.79 = moderate, 0.80–0.90 = strong, > 0.90 = excellent agreement [55].

#### Step 2c: Data extraction

The following data were extracted using a template housed on Covidence: (1) study information (i.e., authors, country of data collection, year of publication), (2) study characteristics (i.e., study design, sampling methods, sample size, methodology, data collection methods, analysis methods), (3) sample characteristics (i.e., age, percent women, type of cancer(s), disease stage, timing), (4) reported yoga background and practice characteristics (i.e., dosage [frequency, length, duration], location, style of yoga, social setting, mode of delivery), (5) conceptual/theoretical approaches, and (6) qualitative findings. If the information presented in the primary article was unclear or missing, the corresponding authors were contacted via email to obtain clarification or missing information (for a maximum of three attempts). To ensure completeness and accuracy in extraction, after JP and SS independently extracted data from all included articles, validation checks were conducted, and JP and SS met to discuss any differences before proceeding to critical appraisal and analysis.

#### Step 2d: Quality assessment

Following the relativist perspective outlined by Sparkes and Smith [56], JP and SS independently appraised the trustworthiness, theoretical considerations, and practical considerations of each study using the Consolidated Criteria for Reporting Qualitative Research (COREQ) 32-item checklist [57]. The checklist was used to assess the quality of included articles based on the inclusion of information pertaining to the research team and reflexivity, study design, and analysis and findings. Article quality was not an exclusion criterion; instead, findings were used to furnish the meta-method analysis and provide insight into the elements of qualitative research that may not be commonly reported in this area of study [58].

### Step 3: Meta-data analysis

For the meta-data analysis, a thematic synthesis approach [58] was carried out by JP and SS. First, JP and SS independently and inductively coded the extracted data that were presented in the “Results/Findings” section of articles line-by-line without any restriction to a prior framework; this was done using concise descriptions that reflected the language used in participants’ quotes or primary authors’ interpretations. When appropriate, the results/findings of the primary articles were coded into existing codes, and new codes were created when necessary. Second, JP and SS met to review the descriptive codes and act as “critical friends” to reflect and explore alternative interpretations and explanations [56], with JB acting as an additional “critical friend” as required to help refine codes. Once codes were agreed upon, JP, SS, and JB sought to create analytical sub-themes. Specifically, the descriptive codes were inductively grouped together based on whether they represented a larger concept or idea related to an aspect of women’s lived experiences. These sub-themes were then grouped together into main themes and categories reflecting broader concepts in the literature.

### Step 4: Meta-method analysis

For the meta-method analysis, data pertaining to the methods used were compared within and across articles [43]. The goal was to determine the frequency of use and identify potential patterns of use. Intervention and program characteristics were also compared within and across studies and are presented briefly in this manuscript for context, with detailed results presented in part II.

### Step 5: Meta-theory analysis

For the meta-theory analysis [43], data pertaining to theory were examined to identify paradigms or ontological approaches that have informed the authors’ theory selection. Potential limitations, strengths, or ambiguities that may influence the use of theory and interpretation of findings pertaining to women’s QoL and well-being after participating in yoga following a cancer diagnosis, and what may have contributed to their experiences, were summarized herein.

### Step 6: Meta-synthesis

By selecting, critically appraising, summarizing, and combining qualitative findings, new interpretations were formed to create an overarching narrative of women’s experiences participating in yoga after a cancer diagnosis, pertaining to part I and II. Also, collating data across qualitative studies enabled conclusions pertaining to: (1) the various methods that have been used to collect and analyze data and (2) potential gaps in knowledge that may be a result of methodological choices in the primary studies.

## Results

### Search results

**Fig. 1:**
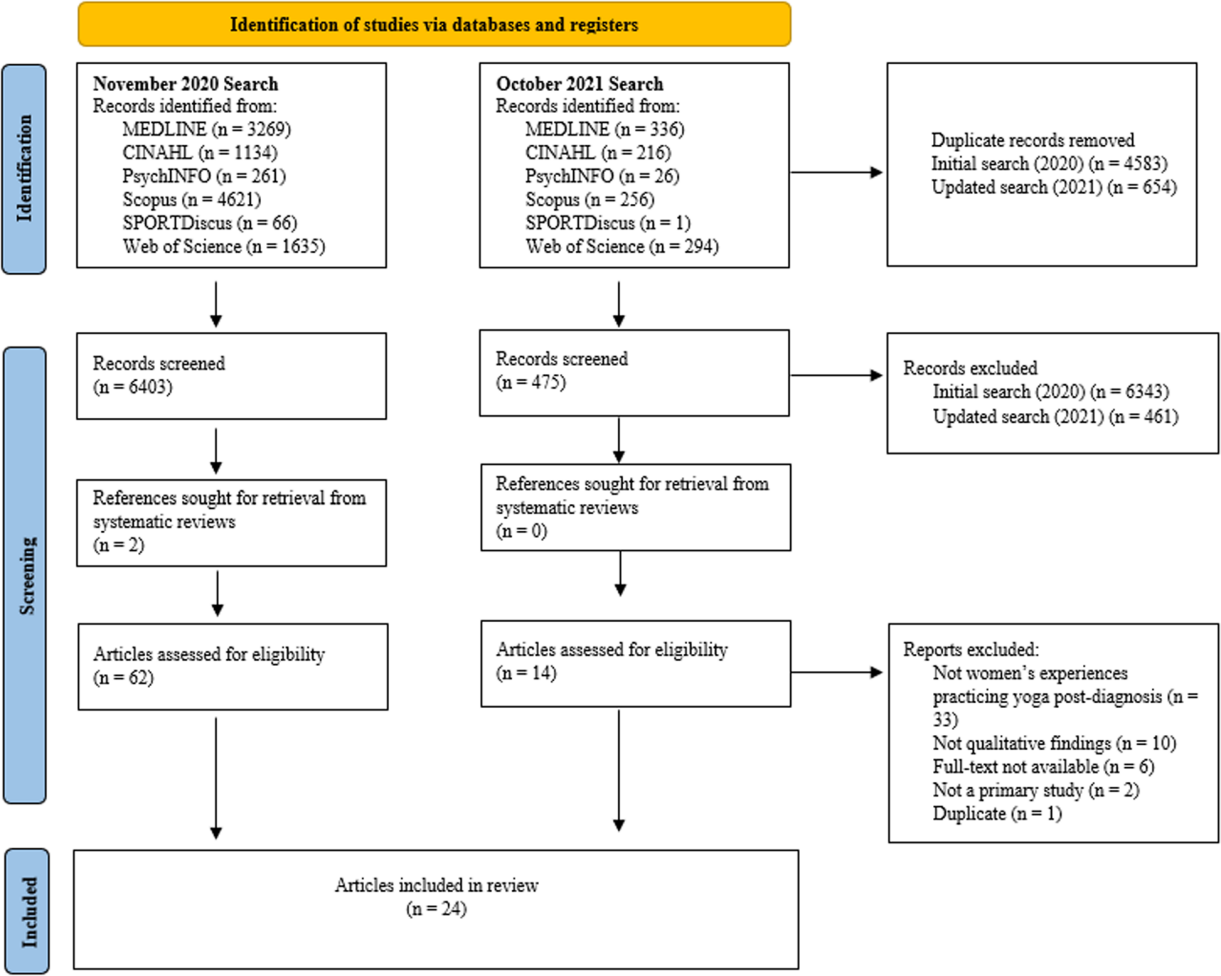
PRISMA diagram of meta-synthesis of women’s experiences participating in yoga after a cancer diagnosis

The database searches identified 12,115 references; 5237 references were identified as duplicates. Using Covidence, 6878 titles and abstracts were reviewed, and 74 full-texts were deemed potentially relevant (Cohen’s kappa = 0.83). These 74 and an additional two full-texts identified during the manual search were screened, of which 24 met the eligibility criteria; the remaining 52 were excluded for the following reasons: did not look at women’s experiences participating in yoga after cancer diagnosis (*n* = 33), did not present qualitative findings (*n* = 10), full-text was not available (*n* = 6), not a primary study (*n* = 2), and duplicate (*n* = 1). Cohen’s kappa at the full-text screening stage was 0.90. The PRISMA diagram of this process is provided in Fig. 1. The characteristics of articles included in the meta-synthesis are presented in Table 1.

**Table 1:** Characteristics of articles included in the meta-synthesis (*n* = 24)

	**First author and year**	**Country**	**Sample**^**a**^	**Intervention**	**Data collection methods**	**Design**	**Analysis methods**	**Theoretical and/or conceptual orientation**
1	Addington 2018 [59]	USA	6 women Treatment status: on-treatment Cancer type: breast Age range: 41–76 Mean age: 59	12 weeks, integral yoga, group-based, synchronous videoconferencing	Program evaluation forms and telephone interviews	Quasi-experimental	NR	NR
2	Archer 2015 [60]	UK	16 women Treatment status: on-treatment Cancer type: cervical, endometrial, ovarian Age range: 31–79 Mean age: 60	10 weeks, Hatha yoga, in-person	Focus groups	Experimental	Interpretative phenomenological analysis	NR
3	Bryan 2021 [61]	USA	7 women, 1 man Treatment status: NR Cancer type: NR Age range: 52–74 Mean age: 61	10 weeks, gentle yoga, group-based, in-person	Semi-structured interviews	Observational	Content analysis	Biopsychosocial-spiritual model
4	Carr 2016 [34]	CAN	3 women Treatment status: NR Cancer type: breast, lymphoma Age range: 60–69 Mean age: NR	3 weeks, Kripaula yoga, 1-on-1, in-person	Open-ended survey questions	Quasi-experimental	Grounded theory approach	NR
5	Danhauer 2008 [62]	USA	51 women Treatment status: on- and off-treatment Cancer type: ovarian, breast Age range: 34–82 Mean age: 58.9	10 weeks, restorative yoga, group-based, in-person	Semi-structured interviews	Quasi-experimental	Categorical aggregation/iterative process	NR
6	Duncan 2008 [63]	CAN	23 women, 1 man Treatment status: on- and off-treatment Cancer type: breast, gynecologic, lymphoma Age range: NR Mean age: 49.3	Community program that was 10 weeks, Iyengar yoga, group-based, in-person	Interviews	Quasi-experimental	Ethnographic content analysis	NR
7	Evans 2017 [35]	NR	3 women, 1 man Treatment status: off-treatment Cancer type: Hodgkin lymphoma, lymphoma, neuroblastoma, breast, osteosarcoma Age range: NR Mean age: 26.5	8 weeks, Iyengar yoga	Journal reflections Weekly phone calls	Quasi-experimental	Content analysis	NR
8	Flanagan 2021 [64]	USA	35 women Treatment status: off-treatment Cancer type: breast Age range: 29–77 Mean age: 56.17	4 weeks, mindfulness-based yoga, self-guided, online asynchronous	Semi-structured interviews	Quasi-experimental	Reflective thematic analysis	Power as knowing participation in change
9	Galantino 2012a [65]	USA	10 women Treatment status: off-treatment Cancer type: breast Age range: 50–71 Mean age: 58	8 weeks, group-based, in-person	Semi-structured interviews	Quasi-experimental	Grounded theory	Social cognitive theory
10	Galantino 2012b [66]	USA	4 women Treatment status: on-treatment Cancer type: breast Age range: 44–65 Mean age: 54.75	12 weeks, Iyengar-inspired yoga, group-based, in-person	Semi-structured interviews	Experimental	Thematic analysis	NR
11	Huberty 2018 [67]	USA	34 women, 5 men Treatment status: on-treatment Cancer type: myeloproliferative neoplasms Age range: 47–83 Mean age: 60	12 weeks, Hatha and Vinyasa yoga, self-guided, online asynchronous	Semi-structured interviews	Experimental	Iterative-thematic approach	NR
12	Kligler 2011 [68]	USA	38 women, 37 men Treatment status: on-treatment Cancer type: NR Age range: NR Mean age: 54.4	1-on-1, in-person	Structured interviews	Quasi-experimental	Thematic analysis	NR
13	Kvillemo 2011 [69]	SWE	18 women Treatment status: off-treatment Cancer type: breast, lymphatic Age range: 31–65 Mean age: 54	8 weeks, Hatha yoga, group-based, in-person	Open-ended survey questions	Experimental	Inductive reasoning approach	NR
14	Loudon 2017 [70]	AUS	15 women Treatment status: off-treatment Cancer type: breast Age range: 36–66 Mean age: 55.2	8 weeks, Satyananda yoga, group-based, in-person	Semi-structured interviews	Observational	Thematic analysis	NR
15	Mackenzie 2014 [71]	CAN	18 women Treatment status: NR Cancer type: breast, other (NR) Age range: NR Mean age: 53.97	1 session, 1-on-1, in-person	Open-ended survey questions	Quasi-experimental	Symbolic interactionism, ethnographic, mindful inquiry, phenomenology	Effort-related attention model, circumplex model of affect, dual-mode theory of affective responses to exercise, neurovisceral integration model
16	McCall 2015a [72]	CAN	11 women, 4 men Treatment status: on-treatment Cancer type: tongue, breast, colorectal-lung, prostate, abdomen, colorectal, skin, brain, blood Age range: 33–72 Mean age: 51.3	Arm 1: 1 session, pranayama-based yoga, group-based, in-person Arm 2: 2 weeks, restorative yoga, group-based, in-person; 4 weeks, mixed yoga, asynchronous website Arm 3: 4 weeks, restorative yoga, group-based, in-person	Semi-structured interviews and journals	Experimental	NR	NR
17	McCall 2015b [73]	CAN	7 women, 3 men Treatment status: on-treatment Cancer type: breast, prostate, bone marrow, skin Age range: 44–71 Mean age: 60.1	Community program with no set number of weeks, restorative yoga, group-based, in-person	Focus groups	Quasi-experimental	Constant comparison technique	NR
18	McDonnell 2020 [74]	USA	16 women, 10 men Treatment status: off-treatment Cancer type: lung Age range: NR Mean age: 66.5	8 weeks, Hatha yoga, group-based, in-person	Focus groups	Quasi-experimental	Interpretative phenomenological analysis	NR
19	Taylor 2018 [36]	USA	26 women Treatment status: off-treatment Cancer type: breast Age range: 33–64 Mean age: 53.75	8 weeks, restorative yoga, group-based, in-person	Focus groups	Observational	Thematic analysis	NR
20	Thomas 2011 [75]	CAN	10 women Treatment status: on- and off-treatment Cancer type: breast Age range: 36–70 Mean age: 52	6 weeks, Iyengar yoga, group-based, in-person	Open-ended survey questions	Experimental	NR	Feminist theory
21	Thomas 2014 [37]	CAN	13 women Treatment status: NR Cancer type: breast Age range: 51–66 Mean age: NR	8 weeks, Iyengar yoga, group-based, in-person	Open-ended survey questions	Quasi-experimental	Content analysis	NR
22	Van Puymbroeck 2011 [76]	USA	18 women Treatment status: off-treatment Cancer type: breast Age range: 33–84 Mean age: 56.67	8 weeks, Hatha yoga, group-based, in-person	Open-ended survey questions	Quasi-experimental	Summative content analysis	NR
23	Van Puymbroeck 2013 [77]	USA	18 women Treatment status: off-treatment Cancer type: breast Age range: 33–84 Mean age: 56.67	8 weeks, Hatha yoga, group-based, in-person	Semi-structured interview guide	Quasi-experimental	Thematic analysis	NR
24	Van Uden-Kraan 2013 [33]	NLD	25 women, 4 men Treatment status: NR Cancer type: breast, colorectal, lung, kidney, brain, endometrial-non-Hodgkin lymphoma Age range: NR Mean age: 53.8	Community program with no set number of weeks, Hatha and Healing yoga, group-based, in-person	Semi-structured interviews	Quasi-experimental	Thematic analysis	NR

*NR* not reported

^a^Female (sex)/woman (gender) was often used interchangeably in the articles; for brevity, woman/women is used in the table

### Quality assessment

**Fig. 2:**
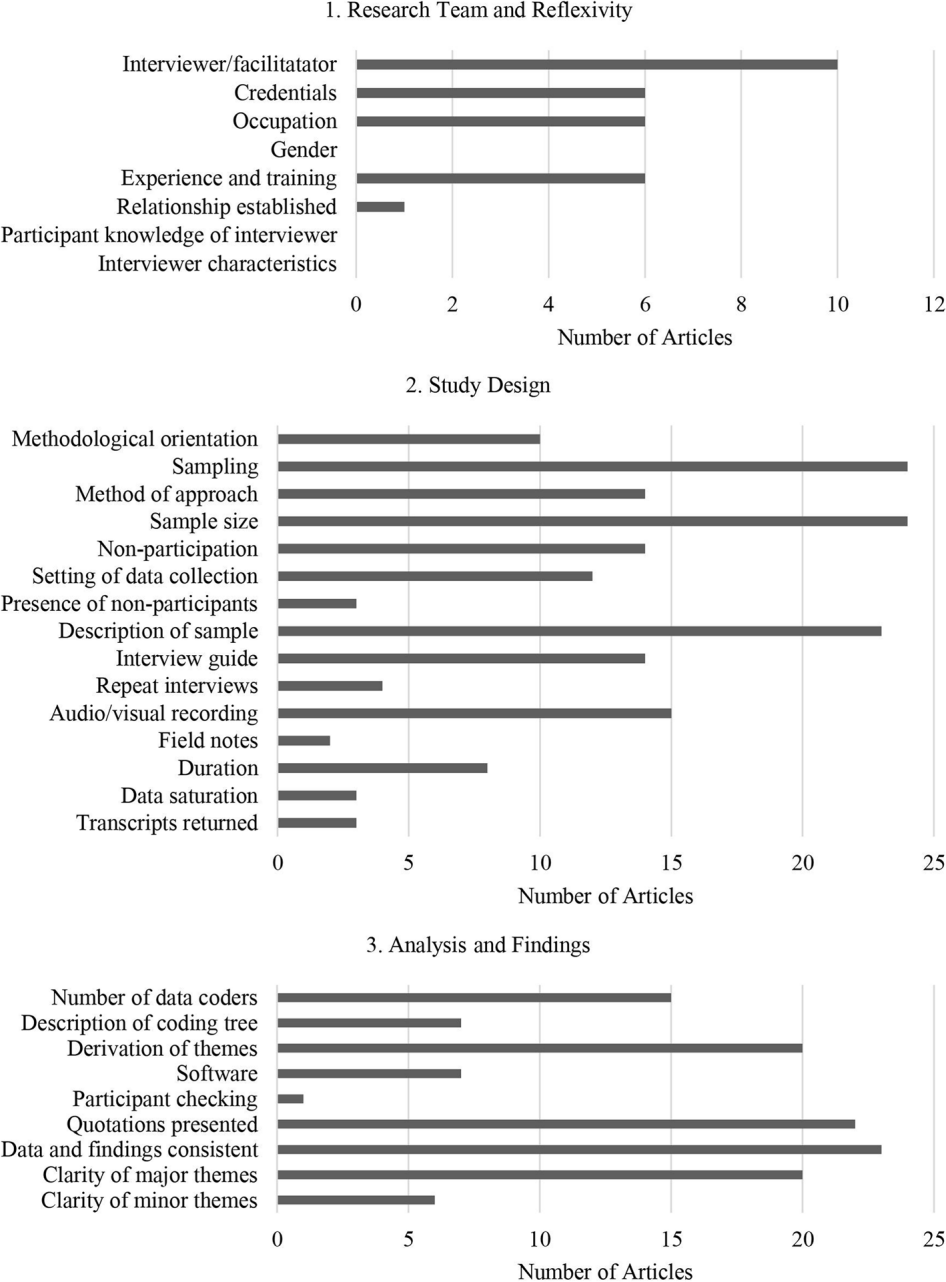
Quality assessment of articles included in meta-synthesis (*n* = 24) using the COREQ Checklist

Domain one (research team and reflexivity practices) was the least reported domain; in this domain, the most reported item was who conducted the interview/focus groups (*n* = 10; 55.6%), and no articles reported on interviewer/facilitator gender and biases or participant knowledge of interviewer/facilitator. Domain two (study design) varied in the level of reporting; in this domain, the most reported items were sample size (*n* = 24; 100%) and sampling procedures (*n* = 24; 100%), and the use of field notes was the least reported item (*n* = 2; 11%). Domain three (analysis and findings) reporting was often high; in this domain, the most reported item was consistency between findings and conclusions (*n* = 23; 95.8%), and participant checking was the least reported item (*n* = 1; 4.2%). See Fig. 2 for an overview of the current reporting practices as per the COREQ checklist.

### Meta-methods results

#### Study information and characteristics

The 24 articles were published between 2008 and 2021. Of the 22 (91.7%) articles that reported on the country where data were collected, seven (29.2%) were conducted in Canada, 11 (45.8%) in the USA, one (4.2%) in the UK, one (4.2%) in the Netherlands, one (4.2%) in Sweden, and one (4.2%) in Australia. For parsimony, study designs were classified into one of the following three categories using the information provided in the articles: (1) quasi-experimental (i.e., delivered an intervention but did not have a comparison group and/or did not randomize participants), (2) experimental (i.e., delivered an intervention, had a comparison group, and randomized participants), or (3) observational (i.e., did not deliver an intervention, natural observation without any attempt to create change). Where explanations of study designs were unclear, a reasonable level of inference was used to identify the most likely study design used. Seventeen (70.8%) articles were quasi-experimental studies, four (16.7%) were experimental studies, and two (8.4%) were observational studies. Also, one (4.2%) article aggregated data from a quasi-experimental feasibility trial and an experimental pilot randomized controlled trial.

#### Sample characteristics

In total, 490 participants were included across the 24 articles, with sample sizes ranging from three to 74 (*M* = 20.4, SD = 16.4). The average age of participants ranged from 26.5 to 66.5 years. All articles specified when participants were recruited; six (25.0%) post-diagnosis (without specification of participants’ treatment status), one (4.2%) post-surgery (without specification of participants’ treatment status), six (25.0%) on-treatment, eight (33.3%) post-treatment, two (8.3%) either on- or post-treatment, and one (4.2%) receiving palliative care. Twenty-two (91.7%) articles reported on type(s) of cancer: 11 (45.8%) mixed diagnoses, 10 (41.7%) breast cancer, and one (4.2%) lung cancer. Thirteen (54.2%) articles reported on cancer stage: eight (33.3%) focused on stages 0 to 3, four (16.7%) on stages 0 to 4, and one (4.2%) on stage 4. Cancer stage was not applicable in one (4.2%) article as it included women with myeloproliferative neoplasms.

#### Yoga intervention and program characteristics

Of the 24 articles reviewed, 23 (95.8%) articles reported results pertaining to a yoga intervention or program. Specifically, 18 (75.0%) articles described one single yoga intervention being investigated, one (4.2%) described three separate yoga interventions being investigated within a single study (i.e., participants received one of three interventions), three (12.5%) described different structured community yoga programs investigated within three single studies, and two (8.3%) described an identical intervention investigated with two single studies (i.e., two articles were published based on the same dataset). Therefore, the final number of yoga interventions or programs described is 25, which was used as the denominator below.

Twenty-four (96.0%) intervention descriptions included mode of delivery: one (4.0%) intervention or program was delivered via synchronous videoconferencing technology, one (4.0%) via a single asynchronously pre-recorded video online, one (4.0%) via asynchronously pre-recorded videos on a website, 12 (48.0%) in-person, and nine (36.0%) in-person with materials to support *supplemental* self-guided practices at home. For studies that included *supplemental* self-guided practices, four (16%) prescribed dosage (i.e., frequency, length, duration), and five (20%) did not prescribe dosage for at-home practices. Not including interventions or programs that provided *supplemental* self-guided practices at home, the social setting of 23 (92.0%) interventions or programs (92.0%) was described, wherein 18 (72.0%) were delivered in a group (i.e., multiple participants with one interactive instructor), three (12.0%) were one-on-one (i.e., one participant with one interactive instructor), and two (8%) were self-guided only (i.e., one participant, no interactive instructor). Details about the location the interventions or programs were delivered (not including *supplemental* self-guided practices at home) were provided for 22 (88.0%) interventions or programs: 14 (56.0%) were delivered in the community, one (4.0%) at participants’ homes, three (12.0%) online, three (12.0%) at a university, and one (4.0%) at a hospital. Interventions and programs lasted 1 to 12 weeks (*M* = 7.3) and lasted 20 to 120 min (*M* = 69.1), ranging from one to seven times per week (*M* = 2.5), yielding a total of one to 56 sessions (*M* = 15.1). The type of yoga delivered was described for 24 (96.0%) interventions or programs: four (16.0%) delivered Iyengar, one (4.0%) Iyengar-inspired, four (16.0%) Hatha, one (4.0%) Hatha and Vinyasa, one (4.0%) Hatha and Healing, one (4.0%) Integral, one (4.0%) Kripaula, five (20.0%) restorative, one (4.0%) Satyananda, one (4.0%) mindfulness-based, one (4.0%) gentle, and one (4.0%) focused primarily on pranayama.

#### Epistemology, methodology, and methods

Philosophical viewpoints were stated in two (8.3%) of the 24 articles and consisted of a pragmatist epistemology in one (4.2%) prospective follow-up study using a one-group, pre-post, sequential-explanatory mixed-methods design and an interpretivist and feminist epistemology in one (4.2%) quasi-experimental study. Of the 10 (41.7%) articles that specified the qualitative methodology used, two (8.3%) cited interpretative phenomenological analysis, two (8.3%) descriptive, one (4.2%) instrumental case study, one (4.2%) mixed-methods, one (4.2%) neurophenomenology, one (4.2%) interpretive description methodology, one (4.2%) community-based participatory research principles, and one (4.2%) a combination of symbolic interactionism, ethnography, mindful inquiry, and phenomenology. Across the 24 articles, 11 (45.8%) used interviews, four (16.7%) focus groups, six (25%) open-ended survey questions, and three (12.5%) a combination of the aforementioned methods as well as program evaluation forms, journal reflections, and weekly phone calls to collect data. Data analysis techniques included thematic (*n* = 6; 25%), content (*n* = 3; 12.5%), grounded theory (*n* = 2; 8.3%), interpretative phenomenological (*n* = 2; 8.3%), constant comparison (*n* = 1; 4.2%), ethnographic content (*n* = 1; 4.2%), summative content (*n* = 1; 4.2%), categorical aggregation/iterative (*n* = 1; 4.2%), iterative-thematic (*n* = 1; 4.2%), inductive reasoning (*n* = 1; 4.2%), reflective thematic (*n* = 1; 4.2%), and a combination of symbolic interactionism, ethnographic, mindful inquiry, and phenomenology (*n* = 1; 4.2%).

### Meta-theory results

Five (20.8%) of the 24 articles reported using theory or models. One used the social cognitive theory to inform their yoga intervention, situating yoga as a means of a simultaneous source of social support and physical activity. Another employed a multi-theory/model approach using the effort-related attention model, the circumplex model of affect, the dual-mode theory of affective responses to exercise, and the neurovisceral integration model to explore affective and physiological responses of yoga; these were used to frame the study, inform data collection, and guide interpretation of the data after inductive thematic analysis. A third stated that their data analysis was informed by feminist theory, though did not explain why or how the theory was used in the quasi-experimental study. A fourth used the power as knowing participation in change to frame the study, inform data collection, and guide deductive qualitative description analysis of data. A fifth used the biopsychosocial-spiritual model to guide the interpretation of the data after inductive thematic analysis.

### Meta-data results

Across the 24 articles, a total of 210 individual codes were generated representing distinct statements. Codes were aggregated into 15 main themes (sub-headings marked in bold italics) with 48 sub-themes (marked in italics in text). The main themes were further coalesced into five overarching categories (sub-headings marked in bold) to organize dominant ideas. Participants viewed yoga as positively impacting their well-being. Moreover, participants’ experiences provide insight into the interconnected and dynamic nature of multiple QoL dimensions and offer understanding of the contextual considerations that may influence QoL dimensions. In addition, participants’ accounts indicate that breathwork and meditation are crucial components of yoga for facilitating QoL. Finally, a small selection of articles provided a starting point for exploring the potential of yoga prompting lifestyle behavior change. Due to the differences in purposes and study designs across articles, not all articles are represented in each category, theme, and/or sub-theme. Below, the categories are presented, along with a description of each theme and sub-theme pertaining to part I. An overview of the themes is presented in Table 2 and supporting data are presented in Table 3.

**Table 2:** Summary of categories, themes, and subthemes identified in the articles included in the meta-synthesis (*n* = 24) related to the changes in dimensions of quality of life

Themes	Sub-themes
*Category 1: Yoga can support improvements in multiple dimensions of QoL in women diagnosed with cancer*	
Regain the ability to perform basic and instrumental activities of daily living	Improved physiological functions of body systems
	Improved physical fitness and body alignment
	Improved execution of tasks or activities
	Improvements in fatigue and energy
	Reductions in pain, numbness, and cancer-related symptoms
Let go of negative emotions and thoughts while embracing inner tranquility	Increased emotional regulation
	Increased experiences of positive feelings, emotions, and mood
Connect the mind and body to gain a deeper self-appreciation and understanding of oneself	Increased mental strength and resilience
	Increased connection to and awareness of the body, behaviors, and capabilities
	Improved coping
	Increase in positive beliefs about the self
	Increased feelings of acceptance, reduced inner critiques, and freeing oneself from negative thoughts, feelings, and beliefs
	Increased understanding of self and increased feelings of empowerment and self-advocation of needs
	Improved outlook and mindset
Quiet the mental chatter to focus on the present	Increased focus
	Increased attention
	Increased feelings of mindfulness
Find contact and connection for emotional support and companionship	Increased sociability
	Feeling connected to others
*Category 2: Women diagnosed with cancer experience an interaction between QoL dimensions*	
Greater control over emotions coupled with more positive emotions supports the functioning of body systems, positive self-evaluations and coping methods, and connection with others	
Improved fitness supports positive self-beliefs, sociability, and emotional regulation	
*Category 3: Elements of yoga that support improvements in QoL dimensions*	
Being in a group of others who share common experiences	
Detailed and tailored instruction	
Physical postures, turning inward, and breathwork	
*Category 4: Breathwork and meditation are integral elements of yoga*	
Separate strategies that can be used in daily life	
Facilitation of inner tranquility, connection to internal states, awareness of body, and decreased rumination on the external	
*Category 5: Yoga practice may support lifestyle behavior change*

**Table 3:** Supporting quotations identified in the articles included in the meta-synthesis (*n* = 24) related to changes in dimensions of quality of life

Theme	Sub-theme	Reference	Sample quotations
*Category 1: Yoga can support improvements in multiple dimensions of QoL in women diagnosed with cancer*			
Regain the ability to perform basic and instrumental activities of daily living	Improved physiological functions of body systems	[33, 34, 37, 65, 67, 68, 74]	“I find that it’s helpful with my breathing because I think part of being anxious about being sick is it messes up your breathing and you catch yourself not breathing fully and properly.” [68]
	Improved physical fitness and body alignment	[33–35, 37, 60, 61, 63, 66, 67, 70, 72, 74–77]	“I am trying more things now. Like, I picked up a box of books and carried them.” [37]
	Improved execution of task or activity	[35, 61, 65, 70, 73, 74, 77]	“I think my balance is a little better. I’m not so afraid to walk fast as I was for a while.” [77]
	Improvements in fatigue and energy levels	[33–35, 61, 63, 65–74]	“My muscles are sore, but I feel more energized.” [66]
	Decreased pain, numbness, and cancer-related symptoms	[33–37, 60, 61, 63–70, 72, 75, 77]	“Less numbness and a general feeling of being closer to ‘normal’.” [37]
Let go of negative emotions and thoughts while embracing inner tranquility	Increased emotional regulation	[33–35, 61, 66, 67, 69, 70, 72, 75, 77]	“I think that it (yoga) helps also in managing work stress too because I’m able to kind of better handle it.” [35]
	Increased experiences of positive feelings, emotions, and mood	[33–35, 59, 61, 62, 65–67, 69–73, 77]	“… it calms my mind and gives me positive energy, especially on a day like today which could turn bad at any moment.” [66]
Connect the mind and body to gain a deeper self-appreciation and understanding of oneself	Increased mental strength and resilience	[33, 77]	“…we stretched [our arms] out and thought about extending our energy [through our fingertips] and it was a lot harder to push our arms down. So I think mentally it showed us that we had strength inside. And that helped me a lot.” [77]
	Increased connection to and awareness of the body, behaviors, and capabilities	[33, 34, 37, 62, 63, 70, 72]	“I’m now more conscious of keeping my body more upright and learning to move in a relaxed manner and by having an awareness of what’s happening with my body trying to maintain a healthy way of being.” [70]
	Improved coping	[33–35, 63, 66]	“I did some breathing and wrist stretches at the eye doctor. It took the stress out of the possibility of surgery.” [66]
	Increase in positive beliefs about self	[33–35, 61–63, 67, 70, 74]	“And we also did a lot of things with our bodies that I didn’t think possible—like hanging upside down and the different ways we stretched. And for confidence, it was great to see.” [35]
	Increased feelings of self-acceptance, reduced inner critiques, and freeing oneself from negative thoughts, feelings, and beliefs	[33–35, 37, 61, 67–70, 72, 74]	“I experience myself being more in my body. And I can connect it to the sense of wellbeing, I feel more empowered due to I can help myself, and [be] more self-accepting.” [34]
	Increased understanding of self, feelings of empowerment, and self-advocation of needs	[33, 34, 37, 62, 63, 66, 67, 70]	“How important it is to take time out of my busy life to check in with my body and emotions on a regular basis.” [62]
	Improved outlook and mindset	[33, 34, 61, 65, 67, 68, 70, 74]	“It’s been very positive. Every time I did it, I would feel really good about myself and about doing it. When you have a chronic illness... it’s really easy to fall into the trap of thinking that’s me. And you lose sight of the fact that there’s a whole lot of you, and I can find a way to tap into that and realize that you’re still strong in ways, it’s really good.” [67]
Quiet the mental chatter to focus on the present	Increased focus	[33, 36, 63, 70, 71, 74]	“I was feeling more focused because my body was doing something, so the mind was not able to go grocery shopping.” [71]
	Increased attention	[70, 71]	“Instead of just doing something, the exercises that we’ve been given, I actually have put some thought into what I’m doing and how I’m doing it, rather than just barging in with no thought at all.” [70]
	Increased feelings of mindfulness	[33, 63, 71]	“Iyengar yoga focuses a lot on the details of the poses and it takes you away from your thoughts; it quiets that chatterbox that’s always going inside your head. It helps you live more in the present and not sweat the small stuff.” [63]
Find contact and connection for emotional support and companionship	Increased sociability	[35, 70, 72]	“The community aspect, we socialize and hike and it’s nice to hear what the other people are doing, I think that’s big factor.” [72]
	Feeling connected to others	[33, 60–62, 65, 66, 69, 70, 74, 77]	“It just was nice hearing [others’] stories and seeing how they were progressing. It felt like I wasn’t alone.” [74]
*Category 2: Women diagnosed with cancer experience an interaction between QoL dimensions*			
Improved fitness supports positive self-beliefs, sociability, and emotional regulation		[34, 35, 66, 67, 69, 70]	“I’m not reluctant to go out any more because I was reluctant in the past to just be active because I didn’t want to be tired all the time afterward. And I’m not so reluctant about that anymore.” [35]
Greater control over emotions coupled with more positive emotions supports the functioning of body systems, positive self-evaluations and coping methods, and connection with others		[34, 35, 67, 69, 70] [35, 66, 70]	“I did feel that every time I did finish one of the sessions like I did really accomplish something and it put me in a better mood and I’m sure that showed when I spoke to people.” [67]
*Category 3: Elements of yoga that support improvements in QoL dimensions*			
Being in a group of others who share common experiences		[33, 60, 66, 69, 70, 74, 75]	“The gathering of the same people with the same condition, that’s really good because everybody understands why each other are here, and so you haven’t got to say or do anything.” [60]
Detailed and tailored instruction		[63, 66]	“I am glad we are practicing balancing, I am awful at it and have found that [Andee’s] instructions really help.” [66]
Physical postures, turning inward, and breathwork		[33, 34, 69, 71, 75]	“When I came in I was drowsy and sleepy. Savasana (supine meditation) is just like having a power nap… at the end of it I was feeling much more energized.” [71]
*Category 4: Breathwork and meditation are integral elements of yoga*			
Separate strategies that can be used in daily life		[33, 60, 65, 66]	“I think I really ought to put half an hour aside and do some real stretching and then I’m busy doing something, but your breathing you can do it anytime, you know, just for the odd 5 min and it just sort of just brings you down.” [60]
Facilitation of inner tranquility, connection to internal states, awareness of body, and decreased rumination on the external		[34, 37, 60, 62, 65–69, 71, 74, 75, 77]	“My greatest joy was in fact working on some of the deep breathing, the meditation breathing, the sort of release to let go, and actually it’s so much more helpful because it keeps you from ending up with that tension headache and the constant knot in the stomach.” [34]
*Category 5: Yoga practice may support lifestyle behavior change*		[35, 60, 64, 67, 74]	“It has just encouraged me to be more active in general…really take stock on a weekly basis of what kind of things have I done and have I been active and often?” [67]

#### Category 1: Yoga can support improvements in multiple dimensions of QoL in women diagnosed with cancer

##### Main theme 1: Regain the ability to perform basic and instrumental activities of daily living

In 22 (91.7%) articles, participants described how yoga positively impacted their physical functioning, which in turn improved overall physical well-being. Participants indicated *improved physiological functions of body systems*, explaining that they felt that yoga contributed to lowered blood pressure [34], improved circulation [67], improved respiration [33, 65, 67], expanded lung capacity [74], decreased shortness of breath [68], reduced constipation [68], and improved arm morbidity for women experiencing lymphedema [37]. Participants also discussed *improved physical fitness and body alignment* as yoga helped them feel more physically fit. Indeed, participants described improvements in flexibility [33, 34, 60, 61, 66, 67, 70, 74, 75, 77], mobility [33, 70], balance [33, 66, 77], stamina [35, 61, 67], strength [33, 61, 63, 67, 70, 72, 75, 77], fitness [63, 66, 77], weight loss and toning [76], and posture [37, 70, 77] and stated that it counteracted physical deterioration [34] and helped them move their muscles [74]. In terms of *improved execution of tasks or activities*, participants described how yoga improved their ability to carry out day-to-day activities [70, 73, 74, 77], engage in other forms of physical activity [35, 61], and return to normal more quickly [65]. *Improvements in fatigue and energy* were reported; participants felt that yoga reduced fatigue [65, 67, 68], increased energy [33, 35, 61, 66, 67, 70, 71, 73, 74], increased feelings of restfulness [61], improved sleep [33–35, 61, 67, 69, 72], decreased insomnia [68], and increased evening sleepiness post-yoga [71] and morning/afternoon wakefulness post-yoga [71]. Also, participants reported feeling physical invigoration [63], rejuvenation [74], and improved vitality [69] post-yoga. Finally, with respect to *reductions in pain, numbness, and cancer-related symptoms*, participants were specific about how yoga alleviated adverse physical disease and treatment effects; namely, it helped through pain relief [33–37, 60, 66–69, 72, 77], reduced numbness [37], reduced stiffness [65, 72], reduced achiness [61, 65], reduced joint pain [61], physical tension relief [63], and eased physical symptoms [77]. More generally, participants described how yoga helped in their recovery from cancer treatment/cancer-related symptoms [33, 34, 64, 65, 67, 70, 75]. However, in one (4.2%) article, participants expressed experiencing pain and discomfort during yoga and found the sequences tiring [67].

##### Main theme 2: Let go of negative emotions and thoughts while embracing inner tranquility

In 16 (66.7%) articles, participants described how yoga positively impacted aspects of their emotional functioning and well-being. *Increased emotional regulation* was a prominent sub-theme; participants felt that yoga allowed them to feel and release negative emotions resulting from the cancer experience [33], increased their emotional stability [61], and improved their ability to manage stressful situations [33, 34, 61, 67, 69, 70, 72, 75], their worries [33, 69], anxiety/anxieties [33, 69], and intrusive thoughts [69]. Also, participants reported that yoga helped decrease stress [35, 61, 66, 69, 77] and feelings of distress [61] and encouraged them to let go of tension, stress, or the need to control a situation [33]. Moreover, in terms of *increased experiences of positive feelings, emotions, and mood*, participants reported increased feelings of relaxation [33–35, 59, 62, 65–67, 70, 71], hope [34], calm [33, 35, 61, 66, 69–71, 77], happiness [33, 67], peacefulness [61, 62, 72, 77], optimism [34], pleasure [34], tranquility [34, 61], positive affect [71], and benevolence [70]. Participants in two (8.3%) articles spoke more generally about how yoga helped improve their mood [67, 73]. However, one participant in one (4.2%) article felt inadequate during yoga [67] and one participant in a different (4.2%) article found it stressful to practice at-home [59].﻿

##### Main theme 3: Connect the mind and body to gain a deeper self-appreciation and understanding of oneself

In 16 (66.7%) articles, participants described personal development of positive psychological attributes through their participation in yoga that contributed positively to their overall psychological functioning and well-being. Regarding *increased mental strength and resilience*, participants described increased mental strength [33, 77], resilience [33], and mental stability [33], as well as feeling more mentally charged/balanced [33]. Participants discussed an *increased connection to, and awareness of the body, behaviors, and capabilities*; participants described a greater connection with the body [37], a mind–body connection [33, 34], feelings of interconnectedness [63], and a restored sense of balance between both sides of their bodies [37]. In addition, participants reported enhanced awareness of body signals [33], physical capabilities [33], bodily awareness [37, 62, 72], understanding of their body [37], awareness of unconscious behaviors [70], and self-confidence to engage in activities of daily living [33]. In terms of *improved coping*, participants described an improved capacity to cope in general [63, 66] and with specific issues (i.e., cancer and treatments [33, 34]; cancer-related outcomes [35]). Furthermore, there was an *increase in positive beliefs about the self*, as participants described increased self-efficacy [35, 63], self-confidence [33, 35, 67, 70, 74], self-worth [67], sense of self [70], and belief in the importance of self-care [62, 63]. Also, participants reported that yoga helped bolster their self-image [67], self-esteem [33], and (re-)connection to their spiritual self [34, 61], and spiritual strength [61]. Another sub-theme was *increased feelings of acceptance, reduced inner critiques, and freeing oneself from negative thoughts, feelings, and beliefs* as participants reported greater bodily acceptance [37], feeling kinder to oneself [33, 69], self-acceptance [34], acceptance of “what is” [72], and cancer-related limitations [33, 37], as well as reduced self-criticism [70], rumination of aches and pains [35, 70, 74], negative body image [70], anxiety symptoms [61, 68], and depressive symptoms [67, 70]. Moreover, as for *increased understanding of self and increased feelings of empowerment and self-advocation of needs*, participants reported that yoga helped them feel more empowered [34, 63, 66], facilitated a process of “turning inwards” to reflect on their needs [33, 62], and helped them become vigilant about their personal needs and encouraged taking steps toward addressing them [70], including being more assertive about their needs [37] and empowered to stand-up for their needs [33]. Participants reported feeling more in control of their health/illness [66, 67] and engaged in self-healing [66] with yoga. Finally, participants reported an *improved outlook and mindset* based on having a more positive mindset [34, 67, 70, 74], an improved outlook [68], a better frame of mind [61], feeling more open [74], increased beliefs about survival probability [33], motivation to engage with life [65, 70], and feeling accomplished post-yoga [67].

##### Main theme 4: Quiet the mental chatter to focus on the present

In seven (29.2%) articles, participants described how yoga positively impacted their cognitive functioning and well-being. With respect to *increased focus*, participants reported that yoga increased their mental focus [33, 63, 70, 71], concentration [33], and ability to think clearly [36, 74]. Participants discussed *increased attention*; they felt their attention shifted away from dissociative attention (e.g., focusing on non-exercise-related stimuli and diverting attention away from internal sensations and present exercise experience) to associative attention (e.g., focusing on internal feedback including breathing rate and muscle soreness) during yoga [71]. Also, participants reported increased attention to their breath [71] and mental awareness [70]. Regarding *increased feelings of mindfulness*, participants reported that their mind stopped wandering during yoga [71] and that they experienced a quieting [63] and calming of the mind [33]. However, some participants in one (4.2%) article did not feel they experienced any improvements in their cognitive functioning [65], and some participants in a different (4.2%) article reported difficulties maintaining focus during yoga [71].

##### Main theme 5: Find contact and connection for emotional support and companionship

In 12 (50.0%) articles, participants described how group-based, in-person yoga positively impacted their social functioning and well-being. In terms of *increased sociability*, participants felt that yoga facilitated interaction with group members outside of yoga sessions [72], improved relationships with others (e.g., partners, friends, co-workers) [35, 70], and led them to feel more sociable [35, 70]. Also, participants reported *feeling connected to others* [61] because group-based yoga (especially when practiced with other cancer survivors) helped them feel recognized [33], understood [33, 60, 66, 69], emotionally supported [33], not alone in their emotions [62], less intimidated to share [77], open to sharing [70], accepted [60, 66, 69, 77], like they belong [70], and less socially isolated [60, 66, 69]. In addition, participants believed that group-based yoga contributed to a sense of co-regulation [69] and camaraderie [65, 74].

#### Category 2: Women diagnosed with cancer experience an interaction between QoL dimensions

##### Main theme 1: Greater control over emotions coupled with more positive emotions supports functioning of body systems, positive self-evaluations and coping methods, and connection with others

Participants provided insight into the potential pathways and connections between the different QoL dimensions and how improvements in one dimension might facilitate well-being in another. In six (25.0%) articles, participants’ improvements in emotional well-being supported psychological, social, and physical well-being. Participants felt that increased emotional regulation in the form of stress reduction helped them develop a more positive mindset [35, 66] and improved their sleep [35, 70]. Experiences of positive feelings, emotions, and mood were reported to help increase self-confidence and sociability [70], improve relationships [67, 69, 70], help to connect to their body and spirituality [34], help lower blood pressure, direct attention away from pain, and rebound from cancer treatment [34]. Still, being unable to control one’s thoughts during yoga did give rise to negative feelings, emotions, and mood for some participants in one (4.2%) article [69].

##### Main theme 2: Improved fitness supports positive self-beliefs, sociability, and emotional regulation

In nine (37.5%) articles, participants reported that improvements in their physical well-being and functioning supported improvements in their psychological, social, and emotional well-being and functioning. Indeed, participants felt that the physical improvements fostered positive beliefs about the self, including increased self-confidence [37, 60, 70, 76, 77], self-efficacy [37], and treatment-related acceptance [76]. Also, participants reported that physical improvements helped them feel better about themselves [34], decreased perceived stress and anxiety symptoms [74], and contributed to improved body image [36, 76]. Similarly, participants described that physical improvements enabled more attention and awareness of their bodies, behaviors, and capabilities, resulting in increased feelings of capability/competence [37, 75, 77], feelings of comfort in their bodies [77], and mind–body connection [34]. Additionally, participants reported less fear to engage in activities [37], feeling better able to challenge preconceived beliefs about their personal limitations [77], and felt more sociable [70] as a result of physical improvements. Finally, participants reported that improvements in mobility helped them to be more open and relaxed in a safe environment [74].

#### Category 3: Elements of yoga that support improvements in QoL dimensions

##### Main theme 1: Being in a group of others who share common experiences

In seven (29.2%) articles, which were all focused on group-based yoga, participants described the presence of others who share common experiences as important for facilitating improvements in well-being. Their presence allowed for sharing their experiences and offering encouragement which supported feelings of acceptance [33, 60, 66, 69, 75], understanding [33, 60, 66, 69, 75], belonging [70], and openness [70]. In addition, seeing others cope with cancer helped participants feel like they could survive their own diagnosis [33] and increased self-awareness of physical abilities [74].

##### Main theme 2: Detailed and tailored instruction

In two (8.3%) articles wherein the intervention was delivered in-person, participants reported that the instructors’ communication style (i.e., language and approach to explaining physical postures) facilitated physical and psychological well-being. Participants felt their improved balance and flexibility were a result of specific and detailed instruction [66]. Moreover, individual-level instruction and the props used in the Iyengar style of yoga were considered to be instrumental in allowing participants to perform postures properly and experience greater self-efficacy [63].

##### Main theme 3: Physical postures, turning inwards, and breathwork

In five (20.8%) articles, participants reported specific elements of yoga that contributed to their cognitive, psychological, and emotional well-being. Participants identified the first supine position (i.e., Shavasana or “corpse” pose) of yoga as contributing to a mental shift (i.e., more focused, attentive, and mindful) because it was associated with sleep states; one participant compared this pose to a “power nap” [71]. Participants were able to relax during the supine meditation and felt re-energized and more awake immediately following this pose [71]. While focusing on the movement and alignment of the body as well as the breath during more strenuous standing sequences, participants felt able to focus and quiet their minds because these sequences required concentration and thus helped them ignore other stimuli [71]. Participants felt that breathwork, relaxation, and working through various postures contributed to feeling mentally balanced/charged [33], increased their coping skills [33], and provided them with stress-management skills [33, 34, 69]. In addition, participants felt that centering the self during yoga led to feelings of optimism, pleasure, and tranquility [75].

#### Category 4: Breathwork and meditation are integral elements of yoga

##### Main theme 1: Separate strategies that can be used in daily life

Although breathwork and meditation can be delivered as standalone interventions, according to yogic philosophy they are integral components of yoga [27]. Of the 24 articles, 15 (62.5%) explicitly mentioned the impact of breathwork and/or meditation on participants’ experiences. Participants felt breathwork required focus, mental effort, and attention [60, 66]. Participants thought of breathwork and meditation as tools or strategies to use beyond yoga sessions because they are easy [60, 66], unobtrusive [60, 66], require little preparation [60, 66], and can be practiced daily [33, 65]. Participants also linked breathwork and meditation to improvements in emotional, psychological, physical, and cognitive well-being.

##### Main theme 2: Facilitation of inner tranquility, connection to internal states, awareness of body, and decreased rumination on the external

Breathwork and meditation supported emotional well-being by increasing positive affect [71]; by facilitating a sense of calm [68, 69], relaxation [62], and peacefulness [68]; and by encouraging a “letting go” stance [34]. In addition, participants felt breathwork and meditation helped them feel more relaxed [66, 77], less stressed [60, 66, 67], and more in control of their reactions in negative situations (e.g., chemotherapy treatment [65]). For psychological well-being, participants felt that breathwork and meditation increased their bodily awareness [60, 66] and helped them manage their anxiety symptoms [67]. For physical well-being, participants reported that breathwork and meditation contributed to pain relief [37], reduced tension [34], increased energy [34], improved sleep [60, 66, 75], reduced coughing [74], and improved lung capacity [74]. Finally, for cognitive well-being, breathwork and meditation helped participants calm their thoughts [67], provided a distraction for maladaptive thoughts [66, 68], and increased focus [71, 77], concentration [71], and awareness of moment-to-moment experiences [69, 71, 75].

#### Category 5: Yoga practice may support lifestyle behavior change

Of the 24 articles, five (20.8%) reported on participants’ perspectives around lifestyle behaviors, including yoga. Participants reported intentions and motivation to continue with yoga post-intervention to sustain benefits [60, 64, 74]. In one article (4.2%), participants expressed an understanding of the need for consistent practice to improve/sustain physical improvements and that they felt hopeful but unsure about their abilities to do so [74]. In addition, participants reported becoming more mindful of their eating behaviors and greater engagement in other physical activities because of yoga. In one article (4.2%), participants attributed behavioral changes pertaining to diet and physical activity to a change in mindset or appreciation for the health benefits of these behaviors; however, not all participants in this article reported these changes [67]. In another article (4.2%), participants described increased physical activity and mindful eating practices stemming from their participation in yoga, which they believed were responsible for their weight loss [35].

## Discussion

Findings from multiple articles presenting qualitative findings can be synthesized to provide an in-depth understanding of the experiences of diverse participants across settings. A systematic overview of all relevant qualitative articles focused on women’s experiences participating in yoga after a cancer diagnosis is lacking as previous reviews have not focused on qualitative research (e.g., [22–25]). Therefore, the aim of this qualitative meta-synthesis was to examine women’s perspectives on the impact of yoga on their QoL and well-being following a cancer diagnosis. Thematic synthesis was used to identify the main, recurrent themes of multiple qualitative articles across settings and subgroups of women with different cancer diagnoses, prognoses, and challenges. The synthesis of 24 articles provided convincing evidence that yoga yields a range of perceived benefits categorized into five broad QoL dimensions that interact: *physical*, *psychological*, *emotional*, *cognitive*, and *social*. The themes and associated quotes across articles showed common experiences and confirm that yoga helps women manage the adverse side effects of cancer and its treatments, rediscover strength and physical abilities, embrace a positive outlook and relationship with themselves, develop strategies for coping with stressors, foster social connections and support, and become more attentive and mindful. The resulting classification of themes in the current meta-study meta-synthesis corroborates current conceptualizations of QoL as a dynamic, multilevel, and complex concept reflecting subjective experience, wherein the multiple dimensions interact [78, 79]. As explored in category 2 of the results, knowing that experiences in one domain (e.g., physical well-being) can influence another (e.g., psychological and emotional well-being) based on women’s accounts is important to ensure QoL measures assess multiple dimensions and that analytical approaches used in future studies capture possible interactions.

Yoga is a mind–body practice that can include meditation and breath practices in addition to guidance on leading an ethical lifestyle [27]. Yet, many of the yoga interventions and programs reviewed in the 24 articles focused primarily on strengthening and stretching the body as well as improving fitness through physical postures (i.e., asanas). Findings suggest that interventions and programs promoting turning inwards for self-reflection, meditation, and breathwork can support participants’ physical, psychological, emotional, and cognitive well-being and should therefore be considered when designing future interventions and programs. Based on the findings, another consideration is to offer women opportunities to connect with others who share common experiences and to engage with an instructor capable of offering detailed and tailored instruction, perhaps through in-person, group-based interventions and programs. A meta-study on the topic of social support and physical activity for cancer survivors asserts this conclusion [80], suggesting group-based yoga may enhance women’s yoga experiences and keep them engaged after being diagnosed with cancer.

This review demonstrates a possible connection between practicing yoga and engaging in other health-promoting lifestyle behaviors such as healthy eating and other physical activities. However, only five (20.8%) articles probed this topic, and even then, they did not provide insight into *why* or *how* such behaviors may be connected to practicing yoga. Perhaps experiencing improved QoL and well-being may have motivated participants to prioritize health-promoting, self-care behaviors/practices to further support their QoL and well-being. Thus, if yoga can support positive behavior change, it may make it even more meaningful for women, especially for those who would like to improve their health and well-being through multiple or holistic means. Future research should seek to understand the underlying mechanisms that foster QoL and well-being in yoga interventions, as well as the association between practicing yoga and behavior change.

### Strengths and limitations of reviewed articles

This review contributes to our understanding of QoL and well-being from the perspective of women practicing yoga after being diagnosed with cancer. Strengths of the included articles were that they: (1) examined the experiences of women with different cancer diagnoses, prognoses, and challenges (although those diagnosed with breast cancer do represent the majority of published work), (2) investigated the different types of yoga, and (3) varied in terms of the social setting, length, dosage, and delivery method of the interventions and programs, as well as presence and delivery of at-home components of the interventions. Although summarizing and analyzing data from multiple, heterogeneous single articles present challenges, it does align more closely with the “real” world and the plethora of yoga classes and programs offered in various communities. It is unlikely that a single yoga intervention or program will be appropriate for all women diagnosed with cancer; therefore, it is necessary to explore women’s opinions on the ideal yoga intervention or program for promoting QoL and well-being to elucidate the most beneficial components. In addition, there was some variation in the qualitative methods used (e.g., semi-structured interviews, focus groups, open-ended surveys); variation in researchers’ lenses helps to provide a more nuanced interpretation of the results. Despite the strengths, there were notable limitations of the primary articles reviewed in this meta-synthesis related to reporting, intervention and program design, study procedures, and conceptualization of QoL.

#### Limitations of reporting

The majority of the reviewed articles did not reference the use of reporting guidelines (e.g., Consolidated Standards of Reporting Trials [81]; Standards for Reporting Qualitative Research [82]), resulting in inconsistent reporting in the primary articles. Relatedly, the authors varied in their descriptions of yoga and research procedures, with some providing very short descriptions. The Checklist Standardising the Reporting of Interventions For Yoga (CLARIFY) guidelines for yoga interventions (i.e., a reporting guideline extension to complement standard reporting guidelines) comprises 21 items across 10 reporting categories [83] and builds on previous recommendations [84]; this can help improve transparency and consistency when reporting yoga interventions or programs. The following section uses the CLARIFY guidelines as a framework for discussing reporting limitations of the primary articles reviewed and can provide direction to researchers and persons seeking to develop yoga interventions and programs.

##### The instructor

The CLARIFY guidelines call for a detailed description of the person delivering the intervention. The results of this review indicate that women find an instructor’s interacting style (i.e., the manner in which an instructor/facilitator interacts with clients or participants [85]) to be a valuable and integral component of how yoga can facilitate QoL and well-being. Fitness and yoga instructors’ interacting styles can significantly affect participants’ enjoyment and affect during/after sessions [85, 86]. Therefore, the authors would benefit from providing as much detail as possible on the interacting style of the person(s) delivering the yoga sessions, including whether they use visual demonstration, verbal guidance, and/or hands-on assistance. Similarly, as yoga instructors have a responsibility to maintain a safe, inclusive, and welcoming environment for participants, the authors should describe how they ensured their yoga instructors were prepared to offer the intervention by detailing their expertise, experience, personal background, and education/training.

##### Content

The CLARIFY guidelines call for detailed reporting of the content of the intervention, including what postures, meditation, and breathwork were delivered. Although findings from this review assert the importance of particular postures, breathwork, and meditation, only three (12.5%) articles provided clear descriptions of the physical postures and only two (8.3%) included details about the breathwork and meditation used. There are nearly 100 “common” physical postures that could be incorporated into a yoga class, each with their own purposes; thus, without a description or breakdown of the intervention, it makes it difficult to replicate the intervention and subsequent findings. Therefore, it is necessary for authors to describe in detail the activities/content of the yoga intervention or program, including the type of yoga and the specific elements practiced (e.g., postures/asanas, breathwork/pranayama, meditation, relaxation), with offered alternatives (if any). Similarly, when and how formal independent practice (e.g., at-home) is recommended as part of the intervention or program should be reported, and copies of materials to support such practice should be made available.

##### Environmental characteristics

Although not a category in the CLARIFY guidelines, the authors describing future yoga interventions or programs should detail the location and layout of the space where the intervention is delivered because environments that have been carefully and effectively arranged may help promote positive experiences. For example, setting a peaceful mood by including elements such as dim lighting, views of nature (e.g., windows in the practice space), high ceilings, and ample practice space can encourage continued practice [86]. In contrast, mirrors in yoga studios have been identified as a source of negative self-perceptions related to one’s physical appearance and capabilities [87, 88]. Having details on the features of the space (e.g., windows, lighting, sound), provision of props (e.g., mats, blocks, straps, bolsters), and arrangement of participants (e.g., circular, side-by-side) would aid those setting out to create an optimal physical environment that supports women’s yoga practice after cancer.

#### Limitations in intervention and program design

Beyond illustrating the need for accurate and comprehensive reporting of yoga interventions and programs to allow readers to understand exactly what has been developed and evaluated, the reviewed articles raise some important questions on the type and dosage (i.e., intensity, frequency, duration) of yoga and combination of content most effective for women after a cancer diagnosis. While yoga interventions and programs prioritized primarily physical postures, limited reporting and heterogeneity across articles make robust conclusions difficult. Nevertheless, findings from this review, coupled with findings from a recent systematic review of meta-analyses of yoga interventions [89], suggest the need to include more components of traditional yoga (e.g., ethical education, postures, breathing, meditation). Future studies should shift away from the tendency to prioritize the physical element of yoga and include more breathwork and meditation, while also seeking to identify the optimal features of yoga interventions and considering the unique needs and preferences of different groups of people who may participate. Explicit comparison of two or more yoga interventions or programs varying in intensities, frequencies, and durations of postures, breath practices, and meditations should be conducted. It may also be prudent to investigate the potential for self-selecting the intensity, frequency, and duration of yoga practices because research in the domain of physical activity suggests that allowing participants in the general population to self-select physical activity can lead to better psychological/emotional functioning [90]. Finally, including the Essential Properties of Yoga Questionnaire [91] would be a valuable addition to study design and reporting to allow researchers to objectively characterize the 14 key dimensions of yoga interventions and programs (i.e., acceptance/compassion, bandhas, body awareness, breathwork, instructor mention of health benefits, individual attention, meditation and mindfulness, mental and emotional awareness, physicality, active postures, restorative postures, social aspects, spirituality, and yoga philosophy) and thus serve to enhance reliability, transparency, validity, and comparison across studies.

#### Limitations of study procedures

While 15 main themes were identified from 24 articles reporting qualitative results from different subgroups of women, those who were middle-aged and diagnosed with breast cancer were overrepresented, indicating a need to closely examine other groups (e.g., young adults, elderly, lung cancer) as this may reveal different QoL and well-being experiences related to yoga as well as different contextual considerations. Second, 19 (79.2%) articles did not report making use of any theory or models. It is unclear why more authors have not anchored their studies in theory or conceptual models, but one hypothesis is that the complex nature of yoga (wherein multiple moving parts of interventions including physical postures, instructors, and peers may influence participants’ experiences) is not well-captured in current theories or models. As such, researchers may need to draw on multiple theories and models that align with the goals of their research or develop theories or conceptual models that support the development of yoga interventions and programs. Last, 21 (87.5%) articles reported on participants’ experiences after completing an intervention; consequently, the conceptualization of yoga and QoL are limited to controlled conditions created by researchers. Studies are needed to confirm if this would also be the case among women diagnosed with cancer who practice yoga independent of an intervention. Finally, studies with longer follow-up qualitative assessments are warranted to investigate the long-term effects of yoga and hence determine if short-term improvements in QoL and well-being are sustained over time.

#### Limitations in conceptualization of QoL

Only four (16.6%) articles reported negative outcomes pertaining to QoL dimensions. While this may occur because of publication bias, this could also be due to bias or oversight in the development of qualitative data collection methods (or true findings); for example, interview questions have a tendency toward eliciting positive feedback. It is important that both positive and negative experiences be assessed and interpreted appropriately in future research. Relatedly, as this synthesis affirms that QoL is multidimensional and dynamic, using flexible data collection methods that allow for exploring change within QoL dimensions and associations between dimensions at various time points (e.g., before, during, and after interventions) would be more in line with the characterization of QoL found in this review. More attention needs to be focused on using diverse qualitative methodologies (e.g., ethnography, narrative discourse, grounded theory) to explore the interconnectedness and multidimensional nature of QoL and well-being after yoga participation. Doing so may help to better understand the processes underlying women’s experiences of their QoL and well-being and thus inform the development of optimal yoga interventions.

### Strengths and limitations of meta-synthesis

The main strength of this meta-synthesis is that a comprehensive and systematic approach following recommendations for a meta-study meta-synthesis was used [43]. Developing the search strategy in consultation with an experienced librarian and having multiple authors independently screen, extract, analyze, and interpret findings from retrieved articles are also strengths. Also, this review adopted a broad approach (i.e., cancer and yoga characteristics were not exclusion criteria) so that data from different subgroups of diverse women (e.g., younger and older age groups) taking part in different types of yoga across different settings (e.g., in-person or online, at a community center, yoga studio, or hospital) could be synthesized to capture all available qualitative data on how yoga impacts QoL and well-being. Similarly, an advantage of including all qualitative articles, regardless of the qualitative methods used (e.g., semi-structured interviews, focus groups, open-ended surveys), is that it ensures that conclusions are not influenced by one specific methodological orientation, allowing for more robust conclusions.

Nevertheless, there are notable limitations that should be considered. First, only peer-reviewed published articles were reviewed, presenting the risk of publication bias (whereby articles showing a beneficial impact of yoga may have been more likely to be published). Second, only articles published in the English language were reviewed. Third, although data analysis was conducted by multiple authors, the themes and subthemes developed herein may differ from those developed by other authors. The authors’ professional and personal backgrounds have influenced the results and interpretation of women’s experiences of QoL and well-being in relation to yoga (much like the values, backgrounds, and experiences of the primary authors of the included articles could have influenced their results). Fourth, studies with < 50% men were included in this meta-synthesis. However, all articles did not report whether quotations or interpretations were based on men’s experiences; therefore, it is possible that some codes may have been derived from men’s experiences. Fifth, most articles in the review focused on asana-based yoga with only one focusing on non-asana-based yoga; the presence of physical movement in the asana-based yoga may have an impact on participants’ experiences. Last, some authors provided very short descriptions/explanations of their data (perhaps, in part, due to journal word count limits) in the primary articles, offering limited insights and potentially contributing less to this synthesis than articles with rich descriptions and comprehensive quotations. Accordingly, the quality of this meta-synthesis cannot be any better than the quality of the individual articles it is summarizing.

## Conclusions

This review used rigorous methods (i.e., meta-study meta-synthesis) to synthesize qualitative research and highlights that yoga can have a positive influence on various dimensions of QoL among women diagnosed with cancer. It represents a much-needed synthesis of this research as prior reviews have focused on quantitative evidence [e.g., [22, 25]]. From the 24 articles reviewed, five overarching categories were identified: (1) women’s experiences of changes in dimensions of QoL (physical, psychological, emotional, social, cognitive), (2) processes related to changes in QoL, (3) contextual considerations for these changes, (4) the importance of breathwork and meditation for improving QoL, and (5) the potential influence of yoga on lifestyle behavior change. Ultimately, findings help extend our understanding of how yoga impacts women diagnosed with cancer. Based on this review, yoga may promote QoL by helping women experience positive physical and mental changes, become more socially connected and supported, and live meaningfully and mindfully. From a research perspective, this review has the specific advantage of synthesizing heterogeneous qualitative findings, making it easier to understand *how* and *why* yoga may support improvements in QoL among women diagnosed with cancer. It also helps generate new hypotheses about intervention/program setting and content, theory, and conceptualizations of QoL; thus, this review allows interested parties to make informed decisions based on all available evidence. In turn, through the process of synthesizing multiple articles, it provides increased confidence that yoga interventions or programs are likely to have significant implications for promoting positive changes in women’s QoL and should therefore feature in future studies and practices seeking to find alternative therapies to promote QoL in this population.

### Supplementary Information

**Additional file 1: Table S1. **MEDLINE Search Strategy.

**Additional file 2: **ENTREQ Checklist.

## Data Availability

Not applicable.

## Abbreviations

QoL: Quality of Life
PRISMA: Preferred Reporting Items for Systematic Review and Meta-Analysis
ENTREQ: Enhancing Transparency in Reporting the Synthesis of Qualitative Research
MEDLINE: Medical Literature Analysis and Retrieval System Online
CINAHL: Cumulative Index to Nursing and Allied Health Literature
MeSH: Medical Subject Heading
COREQ: Consolidated Criteria for Reporting Qualitative Research
CLARIFY: Checklist Standardising the Reporting of Interventions for Yoga
PROSPERO: International Prospective Register of Systematic Reviews

## Footnotes

1 In the context of this manuscript, interventions refer to structured interventions designed by researchers and programs refer to community programs or classes.

2 Female (sex)/woman (gender) was often used interchangeably in the articles; for brevity, woman/women is used in this manuscript.
