# Supplementary material for: Women’s experiences with yoga after a cancer diagnosis: a qualitative meta-synthesis—part I
Source: Syst Rev. 2023 Sep 26;12:176. doi: 10.1186/s13643-023-02350-x (PMC10521480; doi:10.1186/s13643-023-02350-x)
Supplement: Supplementary file 1 — Additional file 1: Table S1. MEDLINE Search Strategy. [file 13643_2023_2350_MOESM1_ESM.docx]

**Supplementary Material**

| Table S1. MEDLINE Search Strategy for article “Women’s experiences with yoga after a cancer diagnosis: A qualitative meta-synthesis – Part I” | |
| --- | --- |
| 1 | (wom?n or female*).ti,ab. |
| 2 | Women/ |
| 3 | Female/ |
| 4 | 1 or 2 or 3 |
| 5 | exp breathing exercises/ |
| 6 | exp yoga/ |
| 7 | exp relaxation/ or relaxation therapy |
| 8 | exp meditation/ |
| 9 | exp mindfulness/ |
| 10 | (yog* or asana or pranayama or dhyana or meditation or relaxation or mindful*).ti,ab. |
| 11 | (breath* adj3 exercise?).ti,ab. |
| 12 | (body adj3 posture?).ti,ab. |
| 13 | (deep* adj3 breath*).ti,ab. |
| 14 | (breath* adj3 technique?).ti,ab. |
| 15 | 5 or 6 or 7 or 8 or 9 or 10 or 11 or 12 or 13 or 14 |
| 16 | exp Neoplasms/ |
| 17 | exp Medical Oncology/ or exp Psycho-Oncology/ |
| 18 | (neoplas* or oncolog* or cancer* or tumo?r or leuk?emia* or carcinoma* or adeno-carcinoma* or lymphoma* or malignan* or melanoma* or metasta* or sarcoma* or adenoma* or adenocarcinoma* or blastoma* or mesothelioma*).ti,ab. |
| 19 | 16 or 17 or 18 |
| 20 | 4 and 15 and 19 |
| 21 | Limit 20 to (English language and humans) |
